# Editorial: Towards a Functional Characterization of Plant Biostimulants

**DOI:** 10.3389/fpls.2021.677772

**Published:** 2021-04-13

**Authors:** Paolo Carletti, Andrés Calderín García, Carlos A. Silva, Andrew Merchant

**Affiliations:** ^1^Department of Agronomy, Food, Natural Resources, Animals and Environment, University of Padua, Padua, Italy; ^2^Laboratory of Soil Biological Chemistry, Department of Soil, Federal Rural University of Rio de Janeiro (UFRRJ), Seropédica, Brazil; ^3^Department of Soil Science, School of Agrarian Sciences, Federal University of Lavras, Lavras, Brazil; ^4^Faculty of Science, School of Life and Environmental Sciences, The University of Sydney, Sydney, NSW, Australia

**Keywords:** humic substances, microorganism, abiotic and biotic stress, protein hydrolysates, seaweed extracts, bacteria

The second United Nations Sustainable Development Goal aims to reach “zero hunger” by 2030. To achieve this, an increase in crop yield is expected while simultaneously increasing the efficiency of non-renewable resource use, protection of soil quality and improvement of agroecological biodiversity. It is therefore necessary and advantageous to learn from nature to develop environmentally friendly inputs and technologies (Perminova et al., 2019). Plant biostimulants are a promising tool to address this issue. A plant biostimulant is any substance, microorganism (or mixture thereof) capable of improving plant nutrition, leading to improved resistance to abiotic stress or food quality traits, independently of its nutrient content (du Jardin, 2015; Rouphael and Colla, 2020). Biostimulants are products that stimulate plant nutrition and growth, commonly acting at low doses. Biostimulants comprise a wide range of compound classes, commonly including (but not limited to) humic substances, amino acids, protein hydrolysates, carbohydrates, algae-derived products, and root growth promoting bacteria. As a result of this broad scope encompassing a wide range of chemical characteristics, characterization of novel compounds with biostimulant activities is frequently reported.

The functional characterization of biostimulants is fast emerging as a new frontier in plant, soil, and microbial research. The synergies, competition and interactions between organisms that take place upon the diversity of chemical and structural components and pools that constitute the phyto-soil system are complex. The magnitude of the challenge to characterize these will require the most advanced tools, technologies and expertise available often in a cross disciplinary context. Ultimately, harnessing the potential benefits of biostimulant function will require a range of approaches from both the fundamental and applied fields of research, in both lab and field conditions. The objective of this Research Topic was to bring together expertise and contributions from around the world to highlight progress and identify future challenges for biostimulant research.

In this special Research Topic of Frontiers in Plant Science, we highlight some of the latest developments in the detection and functional characterization of biostimulants to enhance plant growth and survival to ultimately improve the management of plant, soil and microbial systems. The potential use of biostimulants holds great promise to alleviate plant stress conditions, improve productivity and promote survival whilst in some cases reducing demand for scarce and essential inputs such as fertilizer. To date, the characterization of biostimulants and their complex network of interactions has encompassed a wide diversity of studies, the individual scope of which is determined by the chemical, structural or organismal focus of an observed effect. For example, a chemical elicitor may need to be isolated from a complex chemical matrix. Alternatively, a biological extract may elicit a stress alleviating effect that may also be crop-dependent. All such permutations fall under the development of our understanding of biostimulation and represent progress within this exciting avenue of research. The present Research Topic contains a wide scope of such examples.

Obtaining materials that demonstrate biostimulant activity has cast a wide net, partly to stay within accepted boundaries of sustainability, partly due to economics, but also in an effort to obtain novel chemical mixtures of biological origin. Several authors demonstrate biostimulant activity from a range of biological materials from marine environments such as seaweed extracts (Islam et al.) and coral microbiota (Ocampo-Alvarez et al.) through to terrestrial environments including arbuscular mycrorrhizal fungi (González-González et al.), multispecies microbial biostimulants (Nazari and Smith, Mickan et al.), humic-like substances isolated from lignin rich agro-industrial residues (Savy et al.), soil-derived humic substances (Jindo et al.), rhizospheric organic acids (Macias-Benitez et al.) and even hydrolysed animal protein (Casadesús et al.). Similarly, soil amendments that enhance the chemical and structural complexity of the soil environment, such as the addition of biochar (Tartaglia et al.) show considerable promise and are the subject of significant attention for improve plant growth, fruit yield and affecting gene expression. It is likely that the adoption of such materials by industry requires the identification of chemical entities within these complex matrices that elicit the biostimulant response. Characterizing the chemical composition of such mixtures requires both advanced tools and the metabolomics approach (Savy et al.; Lucini et al.) and in some cases novel biostatistical approaches (Savy et al.) to cope with such chemical and biochemical complexities. Ultimately, if consistent biostimulant effects can be observed across sufficient environmental variation, harnessing the benefits of crude materials such as those demonstrated here may suffice. However, further understanding of the function, mode of action and potential of these materials across a range of environments is, without doubt, a profitable venture.

At the other end of the spectrum, isolating and characterizing specific chemical elicitors of biostimulant activity represents a powerful step toward functional characterization. The isolation of specific chemical elicitors demonstrating biostimulant activity is uncommon. However, several promising candidates such as Omeprazole (Van Oosten et al.), as a stimulant of nitrogen use efficiency and Thuricin 17 (highlighted by both Nazari and Smith and Lyu et al.) as a promising candidate for “second generation” plant growth promoters and biostimulants (e.g., Nazari and Smith) are highlighted here.

The amelioration of stress conditions is a major subset of biostimulant research and highlights the sometimes-subtle distinction between increasing growth and enhancing survival. An array of plant stress conditions are studied in this Frontiers Research Topic reflecting the scope of renewed interest in this discipline. Biostimulant activity is shown to enhance tolerance to the effects of both biotic stress (e.g., *Phytophthora cinnamomi* infection, Islam et al.) to the effects of heat (Carmody et al.), saline conditions (Ocampo-Alvarez et al.), the combined effects of temperature and nutrient deficit (Casadesús et al.) and the combined effects of pathogenic bacteria, growth stage and nutrient deficiency on plant defense mechanisms for precautionary induced expression (Verly et al.). An equally important consideration for biostimulant use is the target tissues/organisms of application to develop a solid framework for targeted use in field conditions. Application of biostimulants and biofertilizers to the seed (Campobenedetto et al.; Dal Cortivo et al.) are shown to have lasting effects not only in growth promotion and tolerance to heat stress, but also in influencing populations of the resultant phytomicrobiome. This, in turn, undoubtedly has a range of downstream effects on the phtyomicrobiome (see for e.g., Dal Cortivo et al.) and modifications similar to latter developmental-stage interventions showing significant promise (see for e.g., Mickan et al.; Lyu et al.; Macias-Benitez et al.; Dal Cortivo et al.; Moradtalab et al.). In addition, small shifts in environmental conditions can have marked influences and confounded effects on biostimulant activity (see Allen and Allen; Lucini et al.) as well as the process of biostimulant extraction and/or processing (Carmody et al.). Only through the characterization of such vulnerabilities in the maintenance and enhancement of biostimulant activity will enable industrial application to prosper.

The property of biological complexity is both a blessing and a curse and highlighted among many studies both here (Lyu et al.; Macias-Benitez et al.; Mickan et al.; Moradtalab et al.) and in the wider literature. The characterization of complex systems must avoid over-simplification, and recognize the beneficial properties of complexity, often imparting multiple nodes of regulation in moderating growth. Interdependencies in chemical and biological components (see for e.g., Macias-Benitez et al.; Moradtalab et al.) are both a major challenge to the reliable application of biostimulants but may also represent a significant benefit in moderating the effects of biostimulant application to enhance survival.

Finally, the demonstration and characterization of sustained biostimulant activity under field conditions is vital to promote industrial-scale uptake for the plethora of potential applications in both production and conservation efforts. For this reason, field application such as that discussed by Lyu et al. and demonstrated by Jindo et al.; Dal Cortivo et al.; and Mickan et al. as well as potential for horticultural application (Casadesús et al.; González-González et al.) are an important advancement in biostimulant research and deserve considerable attention. It is clear that the functional characterization of biostimulant properties and mode of action directly confronts the challenge of understanding highly complex interactions amongst biological entities often among variable environments. Contributions to this field of research, be them incremental or revolutionary, are likely to lead to significant outcomes for industrial application, conservation, ecological intensification and the sustainability of food, forage, fiber, and biofuel cropping systems through the displacement of more resource intensive management tools and practices.

## Author Contributions

AM wrote the first draft. PC, AG, and CS added notes and revised the manuscript. All authors contributed to the article and approved the submitted version.

## Conflict of Interest

The authors declare that the research was conducted in the absence of any commercial or financial relationships that could be construed as a potential conflict of interest.
